# Genomic Insights into Diffuse Intrinsic Pontine Glioma

**DOI:** 10.3389/fonc.2017.00057

**Published:** 2017-03-28

**Authors:** Danielle H. Lapin, Maria Tsoli, David S. Ziegler

**Affiliations:** ^1^Children’s Cancer Institute, Lowy Cancer Research Centre, University of New South Wales, Randwick, NSW, Australia; ^2^Kids Cancer Centre, Sydney Children’s Hospital, Randwick, NSW, Australia

**Keywords:** diffuse intrinsic pontine glioma, pediatric brainstem gliomas, histone H3K27M, ACVR1, PDGFR, preclinical studies, targeted therapies

## Abstract

Diffuse intrinsic pontine glioma (DIPG) is a highly aggressive pediatric brainstem tumor with a peak incidence in middle childhood and a median survival of less than 1 year. The dismal prognosis associated with DIPG has been exacerbated by the failure of over 250 clinical trials to meaningfully improve survival compared with radiotherapy, the current standard of care. The traditional practice to not biopsy DIPG led to a scarcity in available tissue samples for laboratory analysis that till recently hindered therapeutic advances. Over the past few years, the acquisition of patient derived tumor samples through biopsy and autopsy protocols has led to distinct breakthroughs in the identification of key oncogenic drivers implicated in DIPG development. Aberrations have been discovered in critical genetic drivers including histone H3, ACVR1, TP53, PDGFRA, and Myc. Mutations, previously not identified in other malignancies, highlight DIPG as a distinct biological entity. Identification of novel markers has already greatly influenced the direction of preclinical investigations and offers the exciting possibility of establishing biologically targeted therapies. This review will outline the current knowledge of the genomic landscape related to DIPG, overview preclinical investigations, and reflect how biological advances have influenced the focus of clinical trials toward targeted therapies.

## Introduction

Diffuse intrinsic pontine glioma (DIPG) is a pediatric brainstem glioma that originates in the ventral pons, accounts for 75–80% of brainstem tumors in children and has a peak incidence in middle childhood (1–3). Histopathologically, DIPG have been classified as grade II–IV gliomas, namely, diffuse astrocytoma, anaplastic astrocytoma, or glioblastoma (GBM) (4). Under new recently defined WHO classification most would now be defined as diffuse midline gliomas with histone H3K27M mutation, as described below. Patients typically present with a neurological triad of cranial nerve deficits, ataxia, and long tract signs that have manifested over a short clinical history of less than 3 months (1, 5). Diagnosis is ascertained from clinical signs accompanied by the presence of characteristic radiological appearances (3, 5).

Due to the anatomical location of the tumor within the brainstem, they are unable to be resected, and in many centers are not biopsied. Palliative radiotherapy remains the sole standard therapy offered to patients albeit providing only transient improvements to neurological and radiological function (1, 5, 6). Chemotherapy as a neoadjuvant (7), combination (8–15), adjuvant (8–12, 14), and radiosensitizing (15, 16) agent has been extensively explored in over 250 clinical trials. Disappointedly, none of these studies have produced benefit meaningfully superior to radiotherapy, with the median OS ranging from 9 to 12 months (8, 12, 13, 16) and 1-year survival rates of 27–61% (12, 14, 16). The role of MRI in diagnosis, in addition to the concern that tissue biopsy may lead to increased morbidity, has till recently meant that tissue samples have rarely been available for preclinical research, thus hampering biologically driven therapeutic advances (3).

The recent establishment of stereotactic biopsy and autopsy protocols has allowed for acquisition of primary tumor samples that have subsequently facilitated extensive genomic profiling and crucial breakthroughs in key oncogenic drivers (17). Understanding the mutational processes underlying DIPG is of vital importance to identifying critical oncogenic pathways and defining high-frequency mutations with potential therapeutic relevance (18). The identification of biological markers with established targeted agents has transformed the design and direction being undertaken in preclinical and clinical trials with encouraging results already being discovered (19–21). This review will highlight the current state of knowledge of DIPG tumor biology and underlying genomic processes. It will subsequently outline how this understanding is beginning to guide research in both preclinical models and in clinical trials of novel targeted agents.

## The Genomic Landscape of DIPG

Describing the genomic landscape underpinning DIPG tumorigenesis is vital to characterizing key oncogenic pathways and high-frequency mutations that represent actionable targets (18, 22). Pathway analysis of protein and mRNA profiles suggests that DIPG is a unique type of glioma while sharing some biological similarities with supratentorial high-grade gliomas (HGG) such as GBM (18, 23). Despite the highly malignant nature of DIPG, only a limited number of somatic mutations are yet to be implicated as disease defining. The identification of mutations such as histone H3, ACVR1, TP53, PDGFRA, PIK3CA, and MYC highlight, important somatic events associated with tumor evolution (18, 24, 25) (Table 1). However, intra- and inter-tumor heterogeneity has been observed within these disease-defining markers thereby reflecting the inherent barriers toward engendering effective therapeutic strategies (24). Additional somatic aberrations implicated in DIPG include gains in chromosomes 1q (H3F3A), 2q, 8q, and 9q, as well as repeated losses of chromosomes 11p, 17p13.1, 14q, 18p, and 22q (26–29). The discovery of histone mutations, present in up to 80% of DIPG tumors, has revolutionized our understanding of DIPG biology and introduces the potential for redefining clinical and therapeutic management (23, 30, 31). The histone mutation H3K27M is caused by the conversion of a lysine to methionine at residue 27, inducing unique gain-of-function mechanisms that lead to the loss of histone trimethylation (H3K27me3). Reduced H3K27me3 inhibits polycomb repressive complex 2 and promotes abnormal epigenetic silencing (26, 30). Functional analysis has highlighted the role of H3K27M as contributing to abnormal cell-cycle control, inhibition of autophagy and potentially augmenting tumor resistance to radiotherapy (23). However, the precise role of H3K27M in tumor initiation remains undefined as it is not sufficient on its own for tumorigenesis *in vivo* (32). However, the combination of H3K27M with additional mutational events, such as altered cell-cycle regulatory genes (TP53/PPM1D) and growth factor related pathways (ACVR1/PI3KR1), synergistically enhances tumorigenesis and together is thought to be early transformational event in DIPG (26, 32, 33). *In vitro* and *in vivo* models of developmentally relevant neural stem cells with combined histone H3.3, p53, and PDGFR mutations demonstrate increased tumor formation compared to non-mutated counterparts (32).

**Table 1:** **Major genomic mutations identified in diffuse intrinsic pontine glioma**.

Mutation	Incidence (%)	Functional consequence	Clinical outcome
**Main drivers**			
1. Histone H3	80	The hypomethylation of histone H3 proteins, initiated by the conversion of a lysine to methionine residue, produces aberrant cell-cycle function that initiates oncogenesis	↓ outcome vs. non-histone mutated tumors
*H3.3 (H3F3A)*	60–71		Median OS = 9 months ↓ response to radiotherapy ↑ metastasis
*H3.1 (HIST1H3B)*	12–18		Median OS = 15 months ↑ response to radiotherapy ↓ metastasis
2. ACVR1	20–32	Activation of the BMP pathway through the clustering of mutated residues at the glycine/serine enhanced domain	Co-segregate with histone H3.1 mutations ↑ median OS
3. TP53	22–40	Mutated TP53 in the setting of histone H3.3 allows for the evasion of cell death	Co-segregate with histone H3.3 mutations ↑ metastasis
4. PDGFRA	32	Phosphorylation of tyrosine kinase receptors triggers downstream activation of the PI3K and MAPK pathways	Co-segregate with histone H3.3 mutations Enriched proneural expression Clinically aggressive
**Accessory drivers**			
5. PIK3R1/PIK3CA	15	Oncogenes within the PI3K pathway are an obligate partner of histone H3.3 present in clonal populations	↑ angiogenesis ↑ stem cell formation

Histone mutations are subclassified into H3.1 or H3.3 variants, encoded by the HIST1H3B and H3F3A genes, respectively, and include additional novel mutants in HIST2H3C and a lysine to isoleucine substitution (26, 30). H3.1 K27M mutations are exclusively linked to DIPG whereas H3.3 mutations in K27M and G34R/V (a glycine substitution of arginine *or* valine at position 34) are implicated in midline and supratentorial lateral tumors GBM, respectively (26, 34). The clinicopathological variation existing between the H3.1 and H3.3 subgroups, including differing median OS, phenotype, and responses to radiation, reflects previously unrecognized links between DIPG biology and clinical outcome (26, 31). Histone H3.1 is associated with a slight improved survival benefit with a median OS of 15 months and reduced presence of metastasis, whereas histone H3.3 has a median OS of 9 months and an inferior response to radiation therapy (17). Overall, tumors containing the presence of any H3 mutation are associated with poorer outcomes compared with non-histone mutated tumors (19, 26).

ACVR1 mutations have been identified in up to 32% of DIPG tumors, co-segregate with H3.1 mutations, and have been linked to increased median OS (18, 35, 36). Mutated residues cluster around the inhibitory glycine/serine enhanced domain or ATP-binding region and shift the kinase into an active conformation, subsequently leading to BMP pathway activation (18, 35). Somatic mutations in ACVR1 are almost exclusively limited to DIPG with reports by the Catalogue of Somatic Mutations in Cancer highlighting ACVR1 variants as present in only 0.3% of all tumor types (35). Indeed, this would seem highly suggestive of ACVR1 as potential oncogenic driver of tumorigenesis (18). However, germline mutations in ACRV1, including R206H, are present in patients with fibrodysplasia ossificans progressiva who do not proceed to develop DIPG, or other malignancies (35, 37). Nonetheless, as ACVR1 mutations facilitate early tumor propagation in conjunction with other molecular aberrations, they represent novel targets for future therapies (18).

TP53 mutations, corresponding to the 17p13.1 locus, have been identified in 22–40% of tumor samples and frequently occur in the setting of PDGFR amplification (25, 27, 35). TP53 mutations and allelic loss have been reported at comparable rates in both histone H3.3 mutation and wild-type subsets (38). TP53 and to a lesser extent PPM1D mutations represent obligate partners of H3.3 K27M that promote malignancy in DIPG (22). This partnership has specifically been described to evade cell death and senescence by possibly allowing H3.3 K27M to influence epigenetic regulation (22).

Recurring focal gains in receptor tyrosine kinases (RTKs) and regulatory cell-cycle genes in addition to phospho-mammalian target of rapamycin (mTOR) immunopositivity have been reported in DIPG and may also represent valid therapeutic targets (28, 29). PDGFRA is the most commonly observed amplification, present in approximately 32% of tumors, and highlights an important focal gain implicated in the RTK–Ras–PI3K–Akt signaling network, active in 69% of DIPG (18, 22, 27, 29). In pediatric gliomagenesis, PDGFRA strongly promotes phosphorylation at various phosphotyrosine domains, thereby initiating downstream signaling activation of PI3K and MAPK pathways (38). PDGFRA gains and amplifications co-segregate with histone H3.3 mutations have an enriched proneural gene expression profile and are clinically aggressive regardless of histological classification (26, 27, 39).

PIK3R1 and PIK3CA, genes involved in the PI3K pathway, have been recognized as oncogenes present in grade II–IV gliomas including DIPG (24, 25, 39). Mutations in PIK3R1 have been characterized as an obligatory partner in H3.3 K27M and are reported in clonal populations of DIPG. Interestingly, alterations in PIK3CA have been found in subclonal DIPG populations and are regarded as an accessory driver. While PIK3CA mutations are not crucial to DIPG development, they provide an important angiogenic advantage and promote cancer cell stemness (22). While the exact function of PIK3CA in DIPG in clonal and subclonal tumor evolution remains undefined, spatial tumor conservation of PIK3CA further supports the therapeutic advantage of targeting the RTK–PI3K–MAPK pathway (16).

MYC and MYCN aberrations are present in DIPG and constitute transcriptional regulators that specifically enhance gene expression across the whole genome (18). MYCN amplification is associated with hypermethylation, increased histological grade, and chromothripsis at chromosome 2p. The aberrant functionality of these transcription factors further highlights the critical impact of epigenetics within the DIPG mutational landscape (22). Additionally, G_1_ checkpoint regulators CCND1, 2, and 3, CDK4, and CDK6 have been identified as amplified in DIPG, indicating abnormalities in cell-cycle regulation (24).

*In vivo* models have highlighted anatomical and temporal associations between neural precursor cells of the pons and DIPG cells (22). Upregulation of sonic hedgehog (SHh), induces hyperplasia of the ventral pons and may cause precursor cell populations to undergo tumor transformation (22, 23). However, dysregulation of SHh alone cannot induce oncogenesis. Homeobox and HLH genes are associated with brainstem tumors and have a suspected role in reprogramming embryonic signaling organizers during tumor development (27).

## Preclinical and Clinical Development of Targeted Therapies for DIPG

Revolutionary developments in our understanding of the biological processes underlying DIPG have led to the establishment of targeted therapeutic approaches with the aim of clinical translation (19–21, 35). The identification of histone mutations as disease-defining events has led to the investigation of epigenetic modifiers as potential therapies. Recently, panobinostat, an approved multi-histone deacetylase inhibitor, has been shown to have potent anti-DIPG activity (19, 40). Mechanism of action studies showed that panobinostat increased trimethylation and acetylation levels of H3K27M. Panobinostat has been shown to have antitumor efficacy on H3K27M expressing cells *in vitro*, reducing both cell proliferation and viability, and potent activity in some *in vivo* models, but not in others (19, 40). This may relate to a narrow therapeutic index, with dose limiting toxicities seen at the concentrations required to have an antitumor effect (40). A phase I trial exploring the use of panobinostat as a single agent in DIPG is currently underway in North America (http://ClinicalTrials.gov Identifier: NCT02717455).

GSKJ4, a H3K27 demethylase inhibitor, has also been shown to increase H3K27me3 in H3K27M expressing cells at inhibitory concentrations of 1.3–3.0 µM (20) and has demonstrated synergy with panobinostat (19).

An emphasis on the aberrant activation of the RTK–PI3K–Akt pathway in DIPG has led to the investigation of therapies targeting this signaling network (25). *In vitro* use of dasatinib, a multi-tyrosine kinase inhibitor, has demonstrated reduced tumor proliferation due to the down regulation of PDGFRA activity and subsequent damaging of cell-cycle progression at G_1_ (21). The use of temsirolimus, an agent which inhibits mTOR which is a downstream protein of PI3K (41), has been explored in DIPG (42). A phase I study of the concurrent administration of temsirolimus and perifosine, an Akt inhibitor, in pediatric patients with solid tumors including DIPG has tested the concept of dual targeting of the PI3K–Akt–mTOR pathway and shown it to be safe and feasible (42). Currently, an open phase I study is examining the use of temsirolimus with vorinostat and radiation therapy in patients with newly diagnosed and progressive DIPG (http://ClinicalTrials.gov Identifier: NCT02420613). Another trial has been recently opened (BIOMEDE) that is stratifying patients to different RTK–PI3K–mTOR pathway inhibitors based on expression of EGFR and/or loss of PTEN established following stereotactic biopsy. Patients are assigned, and in some case randomized to different treatment arms with erlotinib, dasatinib, and everolimus (http://ClinicalTrials.gov Identifier: NCT02233049).

The use of PD-03332991 (PD), a CDK4/6 inhibitor, has been explored in preclinical models of DIPG where *in vivo* activity was demonstrated in combination with radiotherapy. PD was shown to have greater efficacy in DIPG tumor cells that exhibited Ink4a-ARF loss caused by cytostatic effects of halting progression through G_0_/G_1_ (43). Inhibition of WEE1 kinase, also expressed in DIPG, has been explored as a radiosensitizer and was demonstrated to have antitumor effects *in vitro*, but no activity *in vivo* (44). The use of temozolomide (TMZ), associated with MGMT inactivation and prolonged survival in adult GBM patients, has been investigated in DIPG patients but has failed to yield any therapeutic advantage (9, 10, 45, 46). The inefficacy of TMZ in DIPG patients may result from the poor association of K27M expressing tumor cells with MGMT methylation, present in only 3% of tissue samples (47). Thus, this disparity highlights the importance of biological rationale for driving clinical decision making (48). A recently closed multi-center phase II clinical study has implemented the use of biopsy and molecularly aided determination of treatment with TMZ and or erlotinib based on MGMT and EGFR status (http://ClinicalTrials.gov Identifier: NCT01182350). The trial has completed recruitment with results expected after completion of adequate patient follow-up.

One of the challenges around the development of effective therapies for DIPG relates to the impermeability of the blood–brain barrier (BBB). In fact, the BBB appears to be even more important in DIPG than other brain tumors, as DIPG exhibits reduced BBB permeability when compared to their cortical HGG counterparts (49). This has prompted the exploration of alternative novel delivery methods, such as the use of nanoparticles or distribution of chemotherapeutics *via* convection-enhanced delivery (CED) (19, 50). In the recent years, various approaches have been tested to improve delivery of therapies into brain tumors, such as, polymer/metal based nanoparticles and cell-mediated delivery methods (51). One exciting new delivery method has focused on the use of bacterial cell-derived vehicles to transport chemotherapeutic agents across the BBB (52). These vehicles are conjugated with bi-specific antibodies that can recognize polysaccharide moieties in the bacterial cell wall and EGFR moiety on the other side. Earlier studies have demonstrated by immunohistochemistry that DIPG express high levels of EGFR and reported this to be independent of gene amplification or mutations (28, 53). Preclinical experiments conducted in canine brain cancer models have shown that these EGFR-targeted vehicles loaded with doxorubicin exhibited highly significant tumor regression (54). A phase I study (ECREST) is currently open using mitoxantrone loaded vehicles in patients with solid and CNS tumors (including DIPG) that demonstrate EGFR expression (http://ClinicalTrials.gov Identifier: NCT02687386).

Convection-enhanced delivery represents a novel therapy modality that allows direct administration to local anatomical structures in order to reduce systemic toxicity and to bypass the BBB (40, 50). Administration of the chemotherapeutic agent carmustine indicated enhanced extension of survival in an orthotopic animal of DIPG (55). Small clinical studies and case reports of CED used in DIPG patients have suggested that it is safe and feasible. Anderson et al. reported on the treatment of two children with topotecan administered by CED. Both patients experienced neurological deficits following placement of bilateral catheters. While MRI findings exhibited a reduction in tumor size, the treatment was unable to prolong survival (56). Another study reported on a robotic navigated catheter implantation procedure to direct treatment with carboplatin. The procedure was well tolerated with an objective response initially seen on MRI. However, the patient died due to progression in regions beyond drug distribution (57). An additional study has been performed by the same research group treating a larger cohort of eight DIPG patients with CED of carboplatin. The investigators used multiple catheter implantations that were robotically directed and MRI guided. The patients experienced some neurological side effects following the first treatment but symptoms resolved before subsequent infusions. Preliminary results show that three of eight patients have survived beyond 15 months while seven of eight patients remained alive after a short follow-up interval (58). While these data suggest that the procedure is well tolerated, more advanced data are required to assess patient benefit. CED is also being employed to deliver novel therapies. For example, a currently open phase I trial is exploring the administration of a radioactively labeled antibody known as ^124^I-8H9 for DIPG patients (http://ClinicalTrials.gov Identifier: NCT01502917). ^124^I-8H9 is a chimeric toxin with demonstrated specificity toward B7-H3, a membrane protein that has been recognized as tumor selective in DIPG (50, 59). Conjugation of ^124^I to the anchoring antibody will allow the therapeutic effects of the radionucleotide against glioma cells to extend beyond B7-H3 tumor expressing cells (59). While the feasibility of CED has been established, ongoing studies are helping to improve the instrumentation to determine optimal flow levels and maximize safety and efficacy (50, 56).

The failure of conventional cytotoxic therapies in DIPG has prompted the exploration of alternative therapeutic strategies, such as immunotherapies, that target glioma-associated antigens (GAAs), which are preferentially expressed by tumor cells (60, 61). Immunohistochemical (IHC) evaluation of IL-13Rα2, EphA2, and survivin proteins, three GAAs previously recognized in adult gliomas, has demonstrated expression of at least one GAA in 87% of DIPG samples (60). A small phase I trial evaluating subcutaneous administration of IL-13Rα2, EphA2, and survivin peptide-based vaccinations was well tolerated with preliminary indications of immunologic activity and clinical response (61). The targeting of multiple GAAs is therapeutically advantageous to overcome mixed GAA expression patterns among tumor samples and combat possible immunoediting that may occur within subclonal populations (60, 61). An additional phase I trial is currently exploring the delivery of IL13-PE38QQR, a recombinant mutated *Pseudomonas aeruginosa* toxin, *via* CED for pediatric patients with DIPG and HGG (50, 62). The presence of IL-13Rα2 alone has been recognized in 61–67% of DIPG tumors and is virtually non-detectable in normal brain tissue, therefore making it a suitable candidate for targeted immunotherapy (60, 62). Future trials will likely require pretreatment biopsies to identify biomarkers by IHC in order to appropriately stratify patients based on the expression of GAAs (62).

## Conclusion

The lack of progress of over three decades of clinical trials means that DIPG remains an almost universally fatal pediatric tumor. Improved access to tumor samples for preclinical investigations has led to substantial breakthroughs and the identification of important genomic mutations responsible for tumorigenesis. Moreover, the significant progress toward uncovering targetable mutations has already vastly transformed the preclinical and clinical landscape. Further elucidation of the pathways involved in the growth and development of DIPG will improve our understanding of the biological landscape and by extension provide rationales for novel treatment protocols.

## Author Contributions

DL, MT, and DZ contributed to the initial and subsequent drafts of the manuscript.

## Conflict of Interest Statement

The authors declare that the research was conducted in the absence of any commercial or financial relationships that could be construed as a potential conflict of interest.
